# Photoresponsivity of an all-semimetal heterostructure based on graphene and WTe_2_

**DOI:** 10.1038/s41598-018-29717-8

**Published:** 2018-08-27

**Authors:** Yujie Liu, Chuan Liu, Xiaomu Wang, Liang He, Xiangang Wan, Yongbing Xu, Yi Shi, Rong Zhang, Fengqiu Wang

**Affiliations:** 1 School of Electronic Science and Engineering, Nanjing University, Nanjing, 210093 China; 2 School of Physics, Nanjing University, Nanjing, 210093 China; 3 Collaborative Innovation Center of Advanced Microstructures, Nanjing University, Nanjing, 210093 China

## Abstract

Heterostructures based on two-dimensional (2D) materials have sparked wide interests in both fundamental physics and applied devices. Recently, Dirac/Weyl semimetals are emerging as capable functional materials for optoelectronic devices. However, thus far the interfacial coupling of an all-semimetal 2D heterostructure has not been investigated, and its effects on optoelectronic properties remain less well understood. Here, a heterostructure comprising of all semi-metallic constituents, namely graphene and WTe_2_, is fabricated. Standard photocurrent measurements on a graphene/WTe_2_ phototransistor reveal a pronounced photocurrent enhancement (a photoresponsivity ~8.7 A/W under 650 nm laser illumination). Transport and photocurrent mapping suggest that both photovoltaic and photothermoelectric effects contribute to the enhanced photoresponse of the hybrid system. Our results help to enrich the understanding of new and emerging device concepts based on 2D layered materials.

## Introduction

Heterostructures based on van der Waals (vdWs)-bonded layered materials have opened new avenues for fundamental scientific studies and applied devices^1–4^. The absence of dangling bonds on the surfaces of vdWs materials enables the creation of high-quality hetero-interfaces without the constraints of lattice matching and processing compatibility^5,6^. Indeed, vdWs heterostructures have been realized using different combinations of 2D materials, including metallic graphene^7^, insulating boron nitride (BN)^8^ and semiconducting transition metal dichalcogenides (TMDCs)^9,10^. While interesting electronic and optical properties of these 2D materials are being investigated, peculiar physics at the hetero-interfaces are attracting ever growing attentions^11–14^. Elucidating the interfacial effects and coupling mechanisms for different types of heterostructures and constituents compounds will provide important guidelines for developing functional 2D materials heterostructures.

In addition to conventional semiconductor and metal materials, Dirac/Weyl semimetals are emerging as capable functional quantum materials for optoelectronic and photonic applications^15–21^. Graphene is, strictly speaking, an exemplary 2D Dirac semimetal. Owing to its unique physical properties, *e*.*g*. mono-atomic thickness, universal optical absorption and gate tunable Fermi level, a variety of remarkable optoelectronic devices have been demonstrated by forming different types of graphene heterostructures. For instance, stacking graphene with semiconductors can form a versatile Schottky-type metal-semiconductor junction that is widely used in photodetection, solar energy collection and Schottky diode, *etc*^22–26^. Graphene-hexagonal boron nitride (hBN) heterostructure, as a paradigm of semimetal-insulator junction, offers a huge potential application in the fabrication of atomically thin field-effect transistors, resonant tunneling transistors and diodes^27–29^. In addition, due to the work function difference, graphene is usually doped by 3D metal contact, which can effectively improve the performance of graphene-based photodetectors^30–34^. Although considerable amount of work has focused on graphene heterostructures, graphene-semimetal heterostructures (as well as the other vdWs heterostructures comprised of purely semimetallic constituents) have remained less well studied^35–37^.

In this work, the optoelectronic properties of a hetero-interface formed by semimetal graphene and WTe_2_ is investigated. WTe_2_ is chosen to form an all-semimetal 2D heterostructure as it belongs to an emerging class of Weyl semimetal with significant relevance to optics and photonics^38–42^. In addition to providing potentially better metallic contact than commonly used 3D metals^43^, study of the fundamental properties of such heterostructures may yield new insights into the design and optimization of 2D materials based nanodevices. Here, we study the interfacial transport and photoresponse of all-semimetal graphene and WTe_2_ heterostructure. Multi-fold of photocurrent enhancement, with a photoresponsivity ~8.7 A/W under 650 nm laser illumination, is observed in the graphene/WTe_2_ hybrid device. Transport and photocurrent mapping suggest that both photovoltaic and photothermoelectric effects contribute to the enhancement of photocurrent. Our experimental results, provide new insights in the design of photonic functional vdWs heterostructures.

## Results and Discussions

**Figure 1:**
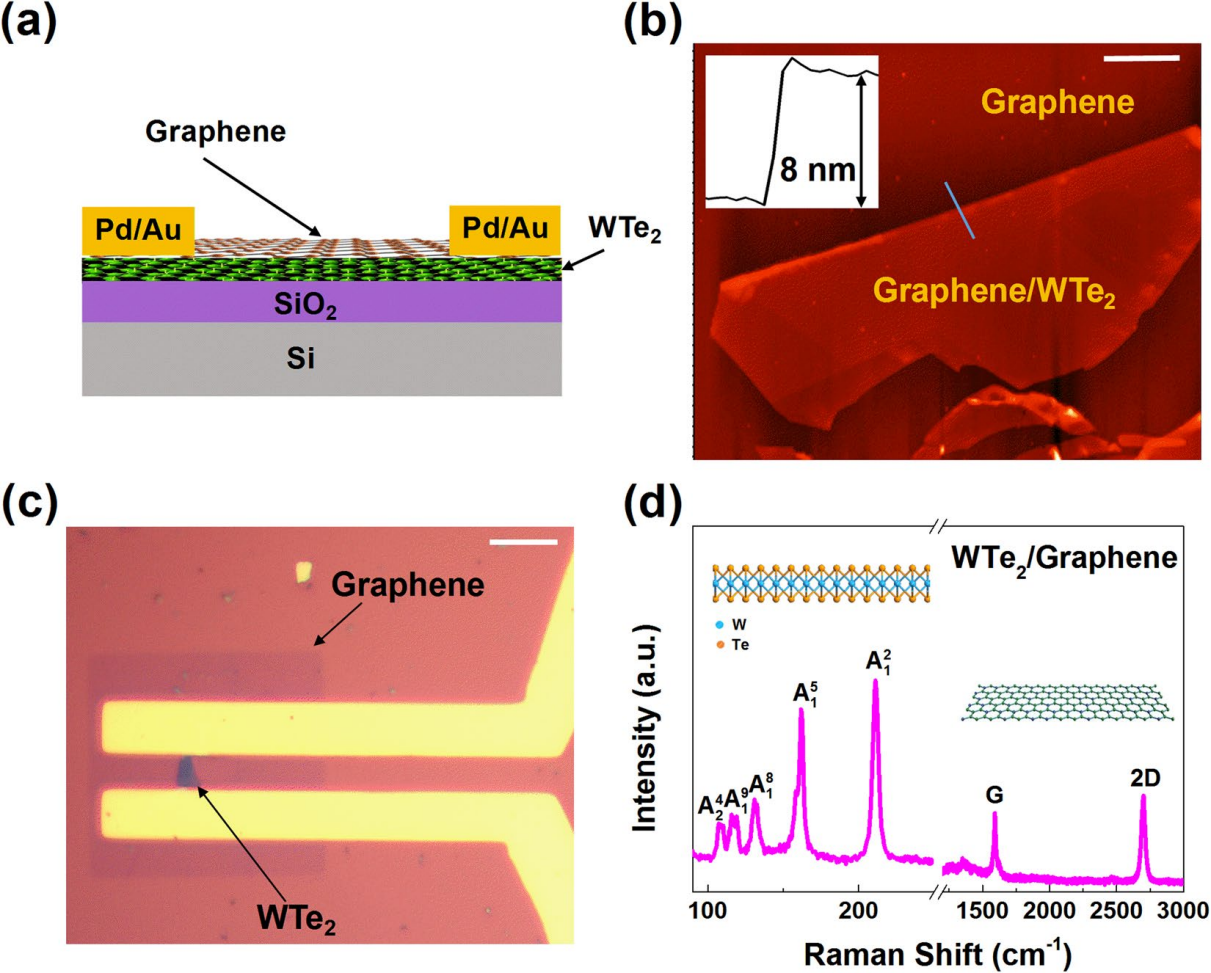
Graphene/WTe_2_ heterostructure device. **(a)** Schematic illustration of the graphene/WTe_2_ heterostructure-based device. **(b)** AFM image of the graphene/WTe_2_ flakes. Inset shows cross-sectional height profile of the WTe_2_ flake on the SiO_2_/Si substrate. Scale: 2 μm. **(c)** Optical image of the fabricated graphene/WTe_2_ hybrid device. Scale: 10 μm. The channel area of graphene/WTe_2_ is 30 × 35 μm. **(d)** Raman spectrum for the graphene/WTe_2_ heterostructure.

Figure 1a schematically illustrates the architecture of the device based on the graphene/WTe_2_ heterostructure. Few-layer WTe_2_ flakes were mechanically exfoliated onto the SiO_2_/Si substrate. Chemical vapor deposition (CVD) grown monolayer graphene was then transferred onto the WTe_2_ flakes using the polymethyl methacrylate (PMMA) supported procedures (see Methods for details). Optical contrast measurements were first used to identify the thickness of WTe_2_ and graphene samples, and further characterizations were conducted by Raman spectroscopy, as shown in Supplementary Figure S1. The results indicate that both samples have good crystalline quality. It should be noted that the Raman features of the WTe_2_ sample well coincide with its Td structure, confirming its semimetallic nature^38,44^. Figure 1b shows the AFM image of the graphene/WTe_2_ stack. From the height profile of the WTe_2_ flake on the substrate, the thickness of it is estimate to be ∼8 nm, corresponding to ~10 layers of WTe_2_. The heterostructure devices are prepared by a standard photolithography procedure (see Methods for details). Since thin WTe_2_ flakes tend to degrade in ambient environment^45^, a fatigue test of the graphene/WTe_2_ film was conducted through optical contrast and Raman spectroscopy, in which the hybrid film showed no discernible change after 11 days of exposure in ambient conditions (see Supplementary Figure S2). The graphene layer is placed on top of WTe_2_, which is envisioned to be effective in mitigating WTe_2_ degradation or protecting inner layers of WTe_2_ from further surface degradation affected by inevitable air exposure during device fabrication (that includes optical inspections to identify the hybrid film, spinning and backing the resist in photolithography to define the metal contacts and the channel, and the lift off process after the metal deposition). Figure 1c shows the optical micrograph of a representative graphene/WTe_2_ device. The graphene channel is about 30 × 35 μm. The quality of the heterostructure device is confirmed by Raman spectroscopy again. As shown in Fig. 1d, the peak positions of both graphene and WTe_2_ agree well with the previous results^38,46^, confirming no significant perturbation to either material during the fabrication processes.

**Figure 2:**
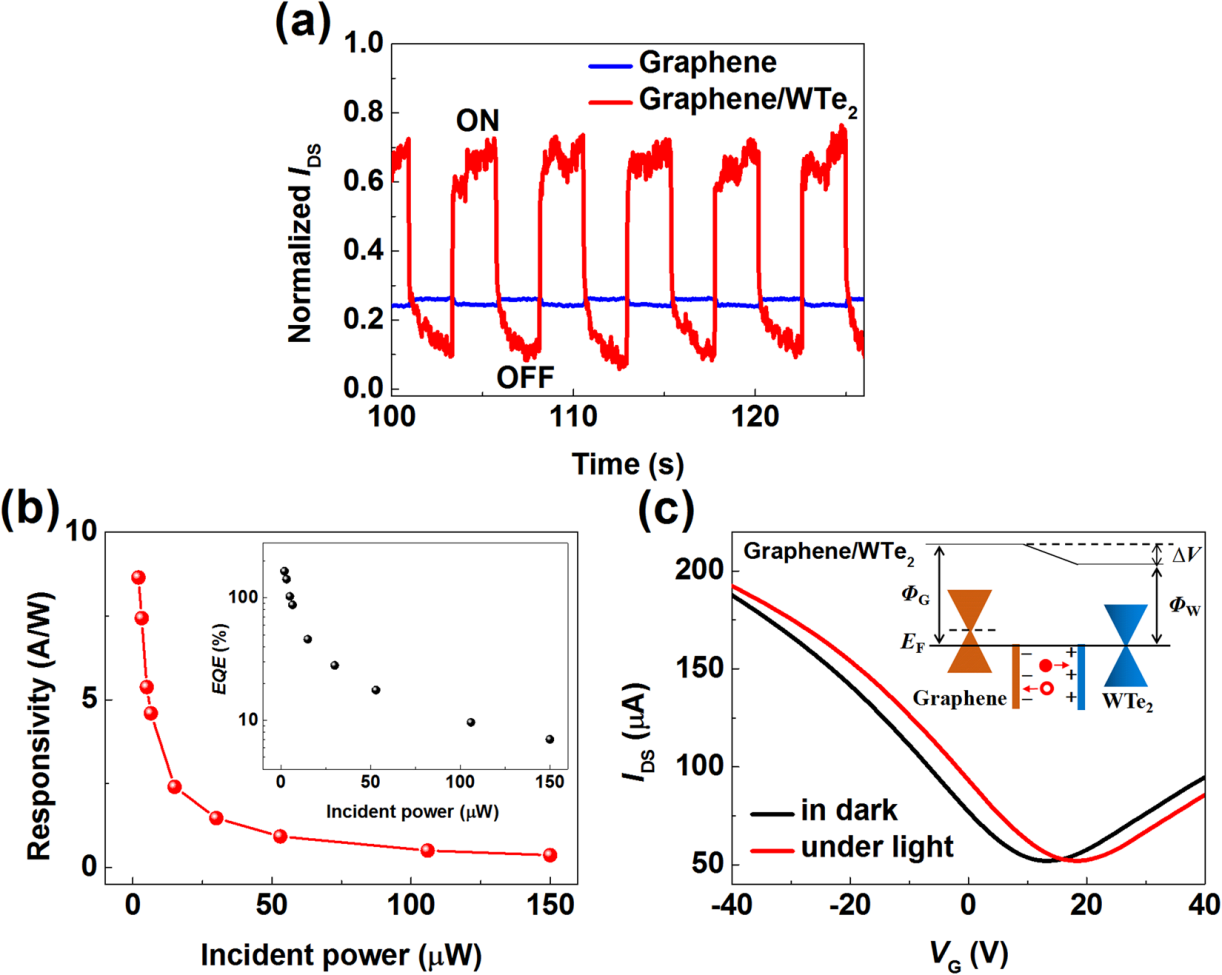
Photoresponse of the graphene/WTe_2_ hybrid device. **(a)** Time-resolved photoresponse of the graphene/WTe_2_ heterostructure device and pure graphene device under 650 nm laser switching on/off. **(b)** Responsivity of the graphene/WTe_2_ phototransistor as function of the incident light power under 650 nm illumination. **(c)** *EQE* of the hybrid device as function of the incident light power under 650 nm illumination. **(d)** Transfer curves of the graphene/WTe_2_ device in dark and under 650 nm laser illumination at *V*_DS_ = 0.5 V. Inset: Schematic diagram of potential step formation and the photo-induced carriers transporting process under light at the graphene-WTe_2_ interface. *Φ*_G_ is the work function of graphene, *Φ*_W_ represents the work function of WTe_2_, ∆*V* is the built-in potential difference. Solid sphere represents photo-induced electron, and hollow sphere is photo-induced hole.

To investigate the optoelectronic properties of the graphene and WTe_2_ heterostructure and to provide insights into the hybridization of the all-semimetal interface, we conducted standard photocurrent measurements with a focused 650 nm continuous wave laser in vacuum (<10^−5^ Torr) at room temperature. Figure 2a shows the normalized transient current response of the graphene/WTe_2_ heterostructure and a pure graphene control sample with the same design (see Supplementary Figure S1). The transient current was recorded as the laser was turned on and off repeatedly at a fixed bias voltage of 0.5 V and the incident power is about 100 µW. As can be seen, the graphene/WTe_2_ device maintains a long-term stability of its current in a series of periodic light stimulations. Moreover, the photocurrent of the heterostructure device demonstrates appreciable enhancement compared with that of the pure graphene device. Generally, Photoresponsivity (*R*) and external quantum efficiency (*EQE*) are the key figure-of-merit that are used to evaluate the performance of a photodetector. *R* is defined as the photocurrent at unit incident optical power and is given by:

1 $$R({\rm{A}}/{\rm{W}})={I}_{{\rm{ph}}}/{P}_{{\rm{opt}}},$$

where *I*_ph_ is the photocurrent and *P*_opt_ is the incident light power on the effective area of the photodetectors. Apparently, high photoresponsivity indicates a large photocurrent can be achieved under relatively low incident illumination power. On the other hand, *EQE* characterizes the effectiveness of conversion of photons to electrons and is defined as:

2 $$EQE=hc{I}_{{\rm{ph}}}/e\lambda {P}_{{\rm{opt}}},$$

where *h* is Planck’s constant, *c* the velocity of light, *e* is the electronic charge and *λ* is the wavelength of the incident power. To compare with other devices, we measured the power-dependent photocurrent of the graphene/WTe_2_ hybrid device at 650 nm (Supplementary Figure S3). The calculated values of *R* and *EQE* as functions of illumination power are plotted in Fig. 2b and its inset. As it can be seen, both *R* and *EQE* present non-linear dependence on the incident light power, and their maximum values appear at low illumination power, which has also been observed in other graphene or MoS_2_ based photodetectors^47–49^. For our specific case, the maximum value of *R* of the graphene/WTe_2_ photodetector is ~8.7 A/W under 650 nm illumination, and the corresponding *EQE* is calculated to be 165% at a low incident power of 2 μW with *V*_DS_ = 0.5 V. These values are about two-three orders higher than the reported ones of pure monolayer graphene photodetectors^50–53^, although they are lower than some of graphene-semiconductor heterostructures^54,55^. Our results for the first time demonstrate that an all-semimetal heterostructure can enable a pronounced photocurrent generation.

To shed lights on the photocurrent enhancement at the semimetal interface, it should be noted that due to the requirement of continuity of Fermi level, the difference in work function between metallic materials (i.e. a conventional 3D metal contact) and graphene can drive a charge transfer process, resulting in an inhomogeneous doping profiles (built-in field) along graphene, which is capable of separating light-induced electron-hole (e-h) pairs. To confirm such a ‘photovoltaic’ picture, transfer characteristics of the graphene/WTe_2_ phototransistor in dark and under illumination were further studied as shown in Fig. 2c. It is seen that the charge neutrality point of the graphene/WTe_2_ hybrid device, upon light irradiation, shifts to a larger positive voltage, unambiguously indicating a hole-doped effect of graphene (or photogenerated electrons transfer from graphene to WTe_2_). Such a shift in transfer curve is compatible with the previously discussed Fermi level pinning effect, when considering the work function for graphene and WTe_2_ are 4.5 eV and 4.39 eV, respectively^30,39^. It should be noted that the rather small work function mismatch (~0.1 eV) would lead to a built-in field that is weak and easily screened by impurity at the interface. The inset of Fig. 2c depicts the likely band bending scenario at the graphene/WTe_2_ interface as stipulated by the difference in work function.

**Figure 3:**
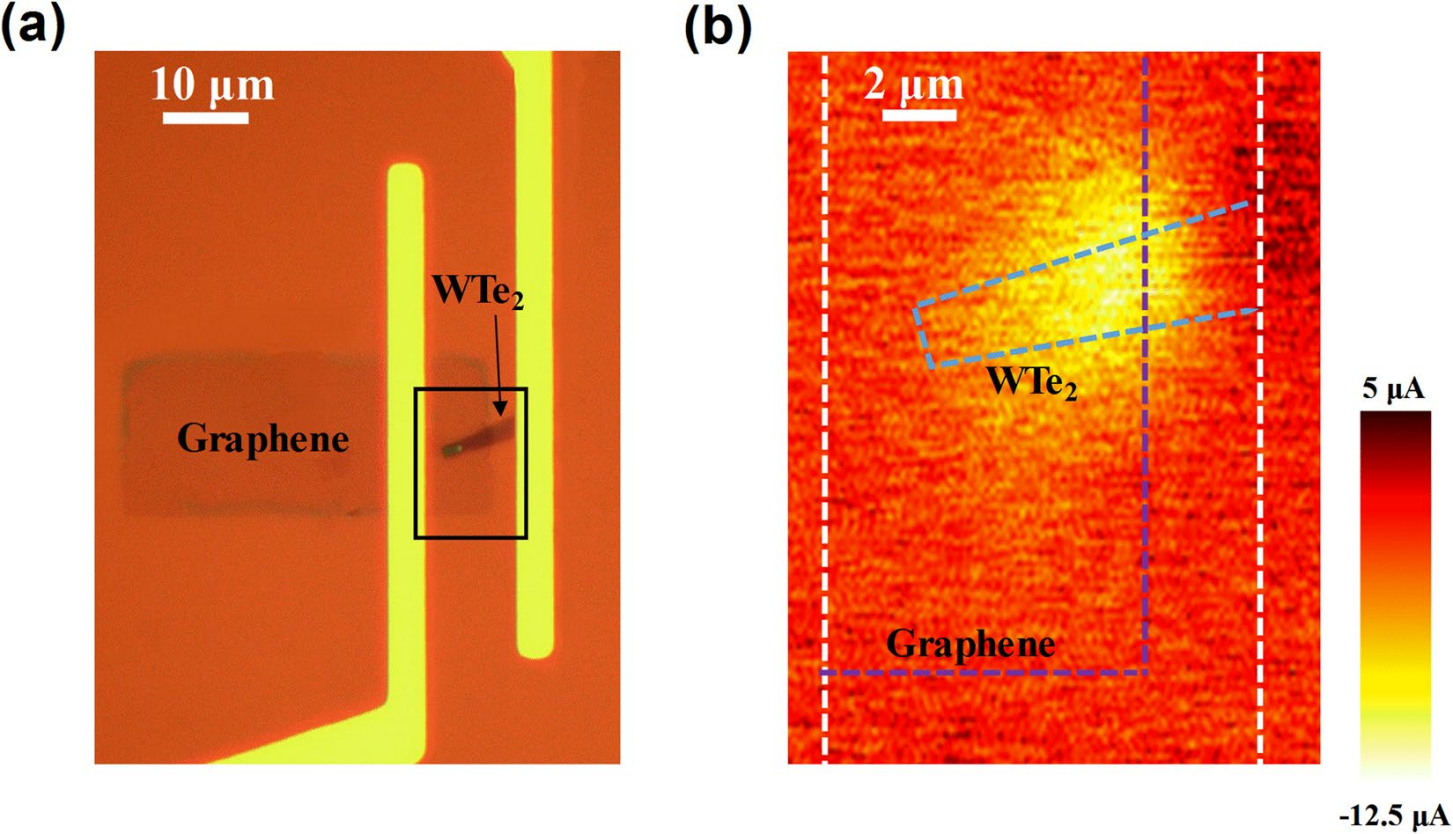
A staggered graphene/WTe_2_ heterostructure device and photocurrent mapping. **(a)** An optical micrograph of the device, with graphene is staggered on top of WTe_2_ flake. The area inside the black square is the scanned area. **(b)** Scanning photocurrent micrograph of the device acquired at *V*_DS_ = 0.1 V, with 0.6 μW power at 532 nm. Regions of photocurrent are observed in the overlapping area outlined by the WTe_2_ flake (blue) with the patterned graphene film (purple). The electrodes are indicated by white dashed lines.

To further elaborate the photocurrent generation mechanism of the graphene/WTe_2_ heterostructure, we fabricated a staggered graphene/WTe_2_ heterostructure device as shown in Fig. 3a. The current-bias voltage (*I-V*) characteristic of the staggered graphene/WTe_2_ device (Supplementary Figure S4) exhibits a slight deviation from the linear relationship, which provides evidence that a potential barrier exists at the graphene/WTe_2_ junction. It should be noted that layer dependent transport measurement has recently suggested the likelihood of the opening of a small positive bandgap when the WTe_2_ film is thinned below 9 nm. Such evolution of electronic structure of WTe_2_ would also facilitate the establishment of a Schottky-like barrier at the graphene/WTe_2_ interface^56^. Complex reconfiguration at the graphene and WTe_2_ interface may also affected the potential barrier, which requires further more detailed investigation. Then we performed spatially resolved photocurrent measurement of the staggered graphene/WTe_2_ heterostructure device under a green laser (532 nm, average power of 0.6 μW). As shown in Fig. 3b, the corresponding photocurrent mapping shows a pronounced photocurrent generation in the overlapping region of the graphene/WTe_2_ junction, which further confirm the important role of the interfacial effects in the heterostructure system. Clearly, the photocurrent enhanced area in Fig. 3b does not exactly coincide with the heterostrcture region. As reported previously, the junction area of graphene due to Fermi level pinning can extend only 200–450 nm away from the metal contact into the graphene channel^32,33^. Therefore, photothermoelectric effect is considered to be another contributing factor to the photocurrent, which results from local heating of the graphene and WTe_2_ junction due to the incident laser^57,58^. On one hand, inhomogeneous doping profiles of the graphene and WTe_2_ junction as mentioned above, give different Seebeck coefficients at the junction. On the other hand, considering graphene’s excellent (up to 5000 Wm^−1^ K^−1^) and WTe_2_’s rather poor (~3 Wm^−1^ K^−1^) thermal conductivities^59,60^, we assume that heat is accumulated in the graphene and WTe_2_ junction when illuminated by light, which leads to a temperature gradient across the device. Then, a thermoelectric photocurrent was induced due to the different Seebeck coefficients and temperature gradient. Considering the small electron capacity and large light-induced changes in electron temperature in graphene, the photothermoelectric effect may play as important a role as in the photovoltaic effect. Investigation of photocurrent generation as a function of layer thickness therefore would yield more insights into the interfacial charge transfer dynamics but is beyond the scope of the current study.

In conclusion, we demonstrated an all-semimetal vdWs heterostructure device through the integration of few-layer WTe_2_ flakes with monolayer graphene. The photocurrent investigation suggests that, despite the all-semimetal nature of the heterostructure (by graphene and few-layer WTe_2_), the interfacial effects can effectively separate the photo-induced electron-hole pairs and lead to pronounced photocurrent enhancement, both due to photovoltaic and photothermoelectric effects. A photoresponsivity ~8.7 A/W is obtained for the heterostructure device under 650 nm laser illumination. These results advance the understanding of fundamental properties of all-semimetal heterostructures and provide guidelines for designing novel photonic devices.

## Methods

### Mechanical exfoliation of WTe_2_

A standard mechanical exfoliation method was employed to isolate few-layer WTe_2_ flakes and transferred them onto a highly doped Si wafer capped by a 285-nm-thick SiO_2_ layer. Thickness of the exfoliated WTe_2_ flakes was measured by means of optical contrast and Raman spectra.

### Transfer of grapheme

A 0.5 cm × 0.5 cm CVD grown monolayer graphene coated with PMMA on copper was immersed in ammonium persulfate for 3 h and deionized water for 30 min, respectively. Subsequently, the graphene film supported by PMMA was transferred onto the WTe_2_ flakes. The PMMA was immediately removed using hot acetone (60 °C).

### Fabrication of devices

The source and drain electrodes (Pd/Au, 10 nm/40 nm) were deposited on top of the graphene/WTe_2_ stacks through a shadow mask by electron beam evaporation. The graphene/WTe_2_ channel was patterned using photolithography procedure and oxygen plasma etching.

### Raman spectroscopy and AFM measurements

The Raman spectroscopy measurements were carried out using a Horiba Jobin Yvon LabRAM HR-800 Raman spectrometer with a 514 nm argon ion laser. Surface morphology of the films was studied using atomic force microscopy (AFM) (Cypher, Asylum Research Inc.).

### Photoresponse characterization

For photoresponse characterization, the 650 nm laser beam is guided through an optical fiber with a FC/PC ferrule and is subsequently incident onto the channel of the devices without focusing. The beam at the device was measured to be Gaussian-shaped with a diameter of about 300 μm. The electrical measurements were conducted using a semiconductor parameter analyzer (Keithley 4200) in a closed cycle cryogenic probe station under vacuum (<10^−5^ Torr) at room-temperature. The photocurrent mapping was performed in a custom data collecting system with a 532 nm continuous wave laser generator, a mechanical chopper, a source meter (Keithley 2612B), and a lock-in amplifier (SR830, Stanford Research Systems Inc.). The transport characterization of the staggered device was measured under vacuum at 24 K for suppressing the outside interference.

## Electronic supplementary material

Supplementary Information
