# Supplementary material for: Photoresponsivity of an all-semimetal heterostructure based on graphene and WTe2
Source: Sci Rep. 2018 Aug 27;8:12840. doi: 10.1038/s41598-018-29717-8 (PMC6110789; doi:10.1038/s41598-018-29717-8)
Supplement: Supplementary file 1 — Supplementary Information [file 41598_2018_29717_MOESM1_ESM.pdf]

SUPPLEMENTARY INFORMATION

Photoresponsivity of an all-semimetal heterostructure based on graphene and WTe_2

Yujie Liu[1,3], Chuan Liu[1,3], Xiaomu Wang[1,3], Liang He[1,3], Xiangang Wan[2,3], Yongbing Xu[1,3], Yi Shi[1,3], Rong Zhang[1,3], Fengqiu Wang[1,3]*

[1] School of Electronic Science and Engineering, Nanjing University, Nanjing 210093, China.

[2] School of Physics, Nanjing University, Nanjing 210093, China.

[3] Collaborative Innovation Center of Advanced Microstructures, Nanjing University, Nanjing 210093, China.

E-mail: fwang@nju.edu.cn

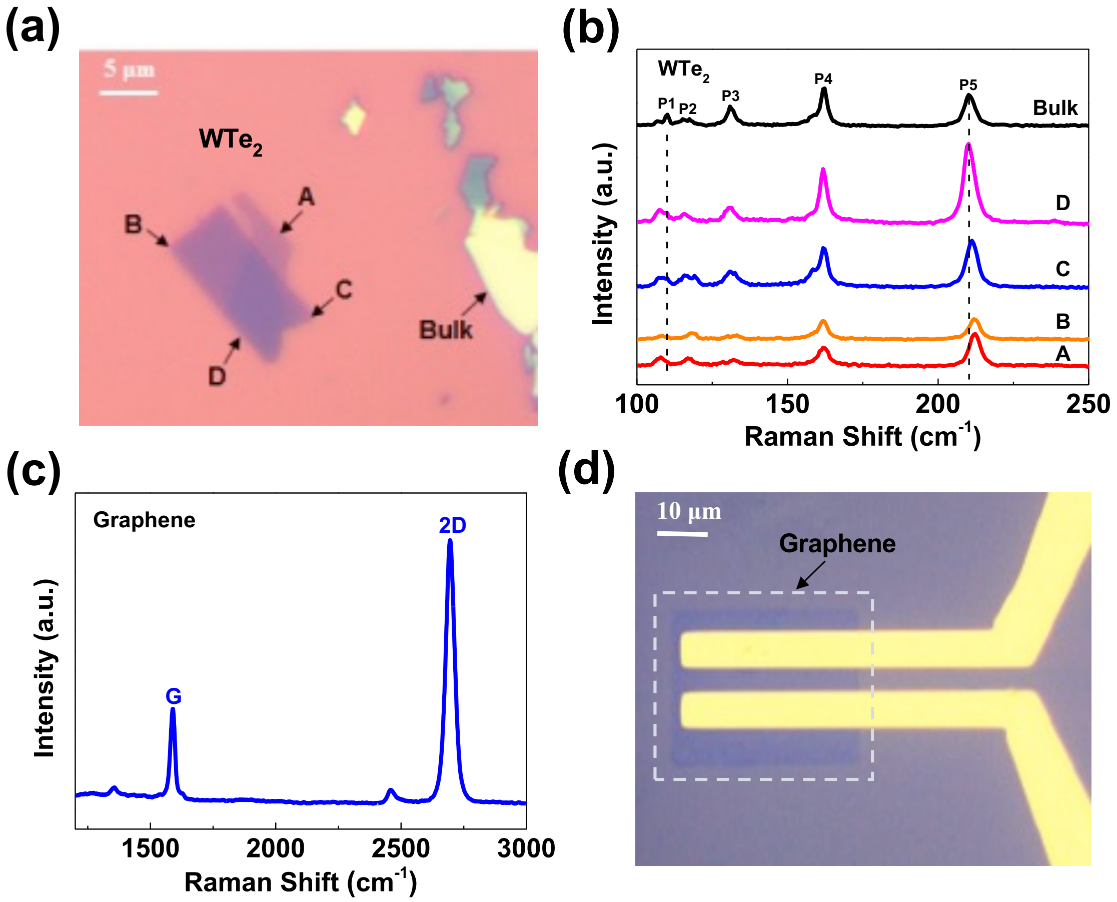

Figure S1. **(a)** Optical image of a WTe_2 sample exfoliated on SiO_2/Si substrate. Optical contrast shows the thickness of WTe_2 flakes is increase from position A to D. **(b)** Raman spectra of bulk and ultrathin WTe_2 flakes in (a). Distinctive peaks, labelled as P1-P5, are clearly observed. As guided by the dotted lines, they follow a systematic shift with increasing the number of layers, the thicknesses of positon A-D are 2~8 monolayers of WTe_2, which is consistent to the previous report [1]. **(c)** Raman spectroscopic characterization of monolayer graphene. It shows a strong 2D peak and G peak, and the Raman intensity ratio of the 2D/G mode is >2, indicating the high quality of our monolayer graphene [2]. **(d)** Optical micrograph of a fabricated pure graphene device. Monolayer graphene as channel is represented in the dashed square. The channel area is the same with graphene/WTe_2 heterostructure device in original text.

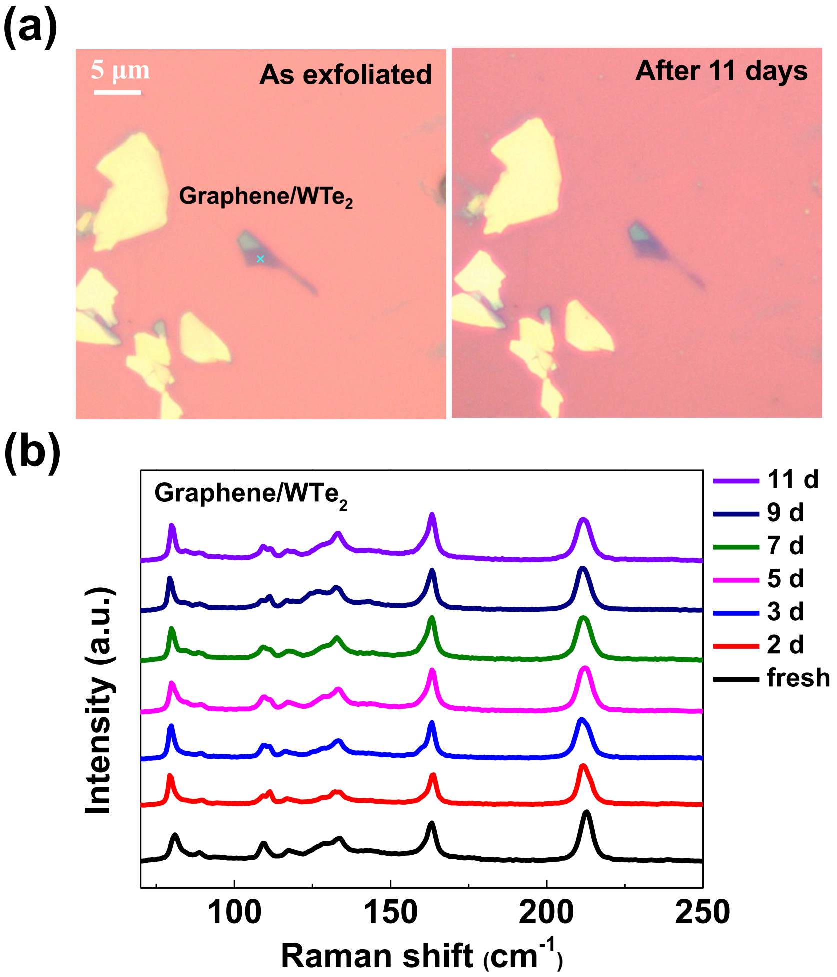

Figure S2. Optical microscope images and Raman spectroscopy results of graphene/WTe_2 hybrid film at sequential stages after its fresh deposition on the Si/SiO_2 substrate under ambient conditions. (**a)** Optical microscope images of fresh graphene/WTe_2 and exposed to air for 11 days. The thickness of WTe_2 flake beneath of graphene is 6 nm. **(b)** Raman spectrum of graphene/WTe_2 hybrid film in (a) during 11 days. Note that the Raman spectrum were recorded at the same region as marked "×" in (a).

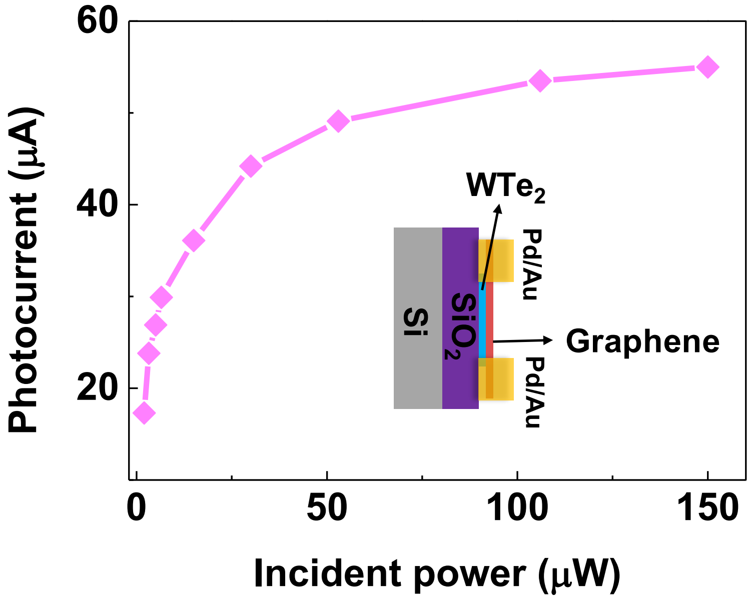

Figure S3. Photocurrent of the graphene/WTe_2 heterostructure device as a function of the incident light power under 650 nm at V_{DS} = 1 V. Inset: schematic of the hybrid device.

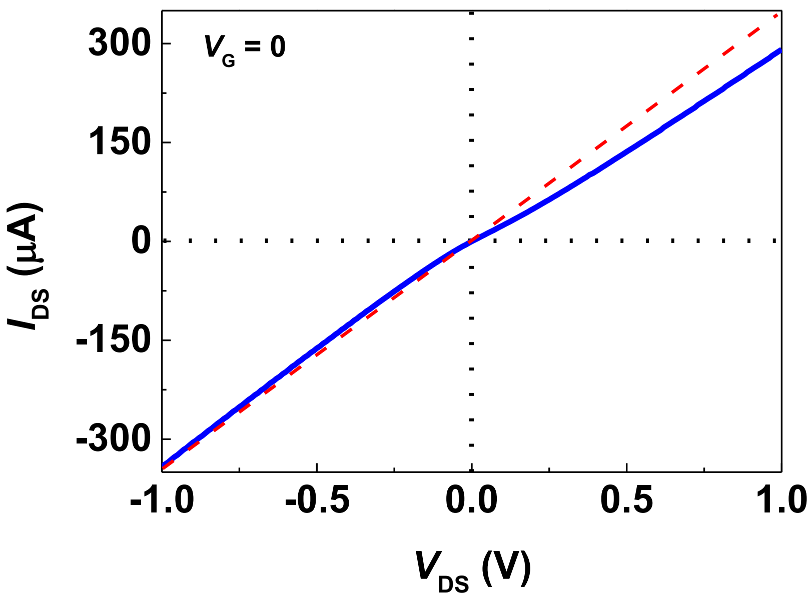

Figure S4. The current-bias voltage (*I-V*) characteristic of the staggered graphene/WTe_2 hybrid device in dark at $V_G = 0$.

1. Lee, J. *et al*. Single- and few-layer WTe_2 and their suspended nanostructures: Raman signatures and nanomechanical resonances. *Nanoscale* **8**, 7854-7860 (2016).
2. Fu, X. *et al*. Graphene/ZnO nanowire/graphene vertical structure based fast-response ultraviolet Photodetector. *Appl. Phys. Lett.* **100**, 223114 (2012).
